# Supplementary material for: Determinants of Working Practice Location for Clinicians According to High School, Medical School, and Resident Training Locations in Korea
Source: Healthcare (Basel). 2023 Apr 22;11(9):1203. doi: 10.3390/healthcare11091203 (PMC10178582; doi:10.3390/healthcare11091203)
Supplement: Supplementary file 1 [file healthcare-11-01203-s001.zip › healthcare-2353126-supplementary.pdf]

healthcare

MDPI

Brief report

Determinants of Working Practice Location for Clinicians According to High School, Medical School, and Resident Training Locations in Korea.

Appendix A

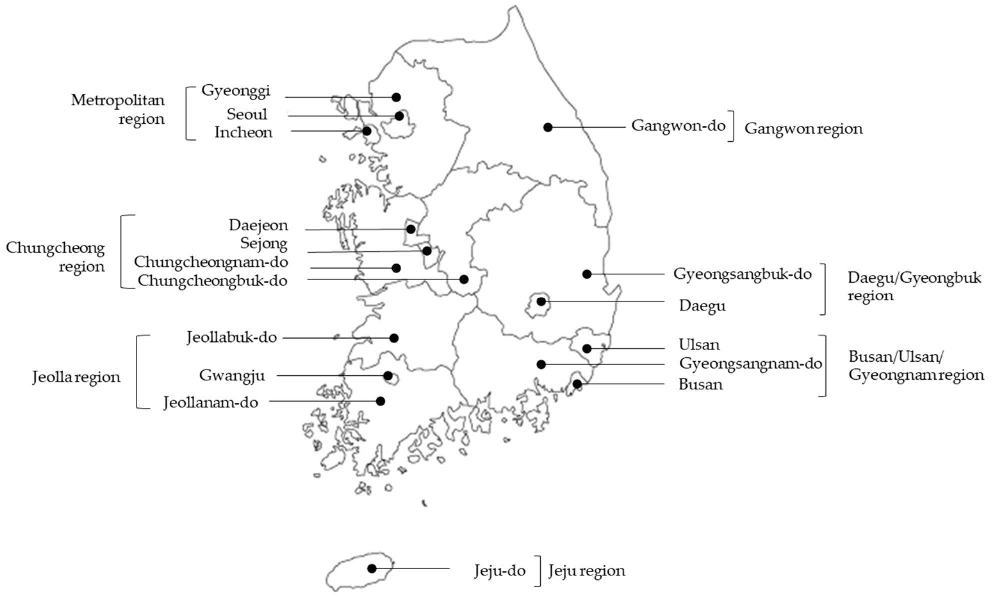

Figure S1. Classified into 7 regions of 17 administrative districts in South Korea.

Table S1. Comparing the number of residents to the number of medical school students in the 7 regions.

		Medical school admission capacity per year (A)*	Resident training Capacity per year (B)†	B/A	Total number of residents ‡	Number of specialists§	Population‖
Total		3,056 (100)	3,374 (100)		9,637 (100)	93,457 (100)	51,439,038 (100)
Metropolitan region	Seoul, Gyeonggi, Incheon	1,118 (36.6)	2,140 (63.4)	1.91	6,176 (64.1)	51,079 (54.7)	25,985,118 (50.5)

non-metropol-itan area	6 regions subtotal	1938 (63.4)	1234 (36.6)	0.64	3,461 (35.9)	42,378 (45.3)	25,453,920 (49.5)
Chungcheong region	Daejeon, Sejong, Chung-cheongnam-do, Chungcheongbuk-do	381 (12.5)	239 (7.1)	0.63	688 (7.1)	8,374 (9.0)	5,547,758 (10.8)
Jeolla region	Gwangju, Jeollanam-do, Jeollabuk-do	485 (15.9)	245 (7.3)	0.51	680 (7.1)	8,799 (9.4)	5,018,354 (9.8)
Daegu/ Gyeongbuk region	Daegu, Gyeongsangbuk-do	350 (11.5)	242 (7.2)	0.69	684 (7.1)	8,348 (8.9)	4,964,183 (9.7)
Busan/ Ulsan/ Gyeongnam region	Busan, Ulsan, Gyeongsangnam-do	415 (13.6)	388 (11.5)	0.93	1,070 (11.1)	13,579 (14.5)	7,708,968 (15.0)
Gangwon region	Gangwon-do	267 (8.7)	95 (2.8)	0.36	268 (2.8)	2,228 (2.4)	1,536,498 (3.0)
Jeju region	Jeju-do	40 (1.3)	25 (0.7)	0.63	71 (0.7)	1,050 (1.1)	678,159 (1.3)

* [2022 Expansion of medical school admission capacity] Admission capacity in 2022 by medical school. Available online: http://www.edujin.co.kr/news/articleView.html?idxno=35873 (accessed on 17 February 2023)

† Recruitment numbers for residents in the first half of 2022 Available online: https://sinim.kha.or.kr/board/nsinim/list (accessed on 17 February 2023)

‡,§: Status of medical personnel by administrative District (Si/Gun/Gu) as of 4Q 2022 Available online: https://kosis.kr/statHtml/statHtml.do?orgId=354&tblId=DT_HIRA4T&conn_path=I2 (accessed on 17 February 2023)

‖: Population by Administrative District (Si/Gun/Gu) and Gender as of Dec 2022. Available online: https://kosis.kr/statHtml/statHtml.do?orgId=101&tblId=DT_1B040A3&conn_path=I3 (accessed on 17 February 2023)

Table S2. Relations between study subgroups and sociodemographic characteristics.

**Variable**			**Total**	**Subgroups**								***p*-value**
				**A**	**B**	**C**	**D**	**E**	**F**	**G**	**H**	
	Total	*n*	7122	311	723	461	3528	272	75	690	1062	
		%	100.0	4.4	10.1	6.5	49.5	3.8	1.1	9.7	14.9	
Sex	Male		5918	269	603	370	2904	242	67	551	912	0.0178
			83.1	4.6	10.2	6.2	49.1	4.1	1.1	9.3	15.4	

	Female	1204	42	120	91	624	30	8	139	150	
		16.9	3.5	10.0	7.6	51.8	2.5	0.7	11.5	12.5	
Age	30-39	1782	63	233	142	793	71	21	187	272	0.0021
		25.0	3.5	13.1	8.0	44.5	4.0	1.2	10.5	15.3	
	40-49	2582	95	262	184	1283	88	31	283	356	
		36.3	3.7	10.2	7.1	49.7	3.4	1.2	11.0	13.8	
	50-59	1886	102	159	112	998	76	21	155	263	
		26.5	5.4	8.4	5.9	52.9	4.0	1.1	8.2	13.9	
	≥ 60	872	51	69	23	454	37	2	65	171	
		12.2	5.9	7.9	2.6	52.1	4.2	0.2	7.5	19.6	
Marital status	Never married	350	10	37	30	157	15	7	47	47	0.4769
		4.9	2.9	10.6	8.6	44.9	4.3	2.0	13.4	13.4	
	Married	6574	295	677	405	3278	245	68	624	982	
		92.3	4.5	10.3	6.2	49.9	3.7	1.0	9.5	14.9	
	Unmarried	198	6	9	26	93	12	0	19	33	
		2.8	3.0	4.6	13.1	47.0	6.1	0.0	9.6	16.7	
Specialty	Medical	3186	133	305	226	1627	126	29	303	437	0.0024
		44.7	4.2	9.6	7.1	51.1	4.0	0.9	9.5	13.7	
	Surgical	2958	132	303	173	1443	113	36	296	462	
		41.5	4.5	10.2	5.9	48.8	3.8	1.2	10.0	15.6	
	Support	978	46	115	62	458	33	10	91	163	
		13.7	4.7	11.8	6.3	46.8	3.4	1.0	9.3	16.7	
Employment status	Own clinic or hospital	3106	168	277	218	1568	123	32	319	401	0.1254
		43.6	5.4	8.9	7.0	50.5	4.0	1.0	10.3	12.9	
	Paid doctor	2712	112	267	188	1255	119	28	282	461	
		38.1	4.1	9.9	6.9	46.3	4.4	1.0	10.4	17.0	
	Medical professor	1304	31	179	55	705	30	15	89	200	
		18.3	2.4	13.7	4.2	54.1	2.3	1.2	6.8	15.3	
